# A survey of genetic and palliative care health professionals’ views of integrating genetics into palliative care

**DOI:** 10.1038/s41431-023-01409-6

**Published:** 2023-06-21

**Authors:** Stephanie White, Erin Turbitt, Kris Rogers, Kathy Tucker, Alison McEwen, Megan Best, Jane L. Phillips, Chris Jacobs

**Affiliations:** 1 Graduate School of Health, University of Technology Sydney, Ultimo, NSW Australia; 2 Hereditary Cancer Centre, Nelune Comprehensive Cancer Centre, Prince of Wales Hospital, Randwick, NSW Australia; 3 Prince of Wales Clinical School, Division of Medicine and Health, University of New South Wales, Sydney, NSW Australia; 4 Institute for Ethics and Society, University of Notre Dame Australia, Sydney, NSW Australia; 5 School of Nursing, Faculty of Health, Queensland University of Technology, Brisbane, QLD Australia

**Keywords:** Palliative care, Genetic counselling, Genetic testing

## Abstract

Genetic counselling and testing have utility for people with palliative care needs and their families. However, genetic and palliative care health professionals have described difficulties initiating palliative-genetic discussions. Between March and July 2022, we received *n* = 73 surveys (6% response rate) from genetic and palliative care health professionals in Australia and New Zealand that assessed and compared barriers and facilitators. The main perceived barrier to both groups was palliative care health professionals’ lack of genetic knowledge (44%). Most palliative care health professionals were ‘not at all confident’ performing several activities, including discussing DNA banking (52%) and knowing their legal responsibilities when sharing genetic information (58%). The most frequently selected facilitator for genetic health professionals was fostering close relationships with palliative care health professionals (52%), while palliative care health professionals indicated a genetic referral template (51%) would be of assistance. Almost all participants agreed genetic discussions do not undermine the central ethos of palliative care (87%). Fewer palliative care health professionals considered themselves well situated to have genetic discussions with a palliative patient’s family compared to genetic health professionals (*p* = 0.014). Our results suggest that genetic and palliative care health professionals support integrating genetics into palliative care, although refinement of the palliative care health professionals’ role in this process is required. We have identified intervention targets to overcome barriers related to knowledge and confidence, which ought to be integrated into future interventions designed to support health professionals deliver the benefits of genetic information to people with palliative care needs and their families.

## Introduction

Genetic counselling and testing can yield important information for individuals and families at all stages of life [1]. In the palliative care setting, the clinical benefit is predominantly for relatives, rather than the patient having testing. Providing a patient who has palliative care needs with the opportunity to engage in genetic counselling (and if indicated, DNA banking or testing) can ultimately help family members to assess and manage future disease risk by, for example, engaging in recommended screening or risk-reducing surgery [2].

Additionally, patients with palliative care needs may experience personal or psychological benefits beyond those related to clinical intervention. Addressing patients’ pre-existing concerns about genetic risk may resolve unmet psychological needs, assist in making meaning from their illness, provide reassurance that family members are receiving relevant information, and support altruistic motivations to help others [3–6]. Despite these benefits, several barriers (discussed further below) and a lack of evidence-based support prevent genetic and palliative care health professionals from initiating discussions about genetics with patients who have palliative care needs [7]. Developing a robust evidence base will tailor support for health professionals to identify patients eligible for genetic testing and provide genetic counselling before the patient dies and the opportunity to gather genetic information is lost. In so doing, family members will have better access to predictive genetic testing and a more personalised genetic risk assessment [8].

As the demand for genetic counselling and testing increase, so too does pressure on existing genetic services [9]. Targeted efforts to improve the capability of ‘non-genetic’ health professionals aim to improve access and delivery of genetic services to patients and families [10, 11]. An understanding of the barriers and facilitators relevant to each context will support the development of appropriate interventions [12]. In the palliative care context, the small body of evidence about the barriers and facilitators leaves several gaps [13]. For example, a commonly reported barrier is low genetics knowledge and confidence amongst palliative care health professionals [4, 14, 15]. These descriptions (i.e., ‘low confidence’) are not specific enough to inform the development of an intervention to support palliative care health professionals in the areas in which they feel deficient. By defining the activities requiring support, directed interventions can be implemented for the greatest impact [12].

Another gap is found in the evidence describing genetic and palliative care health professionals’ views of the potential harms and benefits of genetic discussions [4, 16–21]. Qualitative studies have begun to illustrate the motivations underlying health professionals’ decision making in the palliative context, such as the possibility for genetic discussions to cause psychological harm to patients and families. To advance our understanding, themes about harms and benefits should be examined with quantitative methods to determine whether reported attitudes are generalisable. However, to our knowledge, there does not appear to be any triangulation of the qualitative descriptions of harms and benefits [14, 15, 22].

Another area requiring further examination are reports from health professionals that the palliative care context is an ‘inappropriate’ place to discuss genetics [20]. Other work places less emphasis on the appropriateness of the palliative care setting and tends to report palliative care health professionals’ uncertainty about the role they play in addressing genetics [16, 23]. Additionally, there is a scarcity of evidence from the genetic health professional perspective about whose role it is to broach and facilitate genetic counselling and testing for patients with palliative care needs [17]. Eliciting genetic and palliative care health professionals’ views about their role in integrating genetics into palliative care may enhance the provision of genetics to this population.

To fill these gaps and further existing knowledge about the barriers and facilitators, we aimed to assess and compare the experience, confidence, and attitudes of genetic and palliative care health professionals towards addressing genetics with patients who have palliative care needs and their families.

## Materials and methods

We designed a quantitative, cross-sectional survey study that assessed genetic and palliative care health professionals’ views towards integrating genetics into the care of people with palliative care needs and their families. We took a broad approach to defining a ‘palliative care’ setting, by including any setting in which palliative care could be delivered (e.g., community, hospital, hospice). A study protocol is available on the Open Science Framework (https://osf.io/n6dfh). Reporting items align with the STROBE statement [24]. We did not hold a priori hypotheses as this was an exploratory study with limited theoretical or empirical data available on which to base predictions.

### Participants and recruitment

Participants were eligible if they were (a) a palliative care health professional or (b) genetic health professional. We defined health professionals as medical doctors, registered nurses, and genetic counsellors. To ensure responses were relevant to clinical practice, participants were required to be currently, or have previously, worked in a clinical area.

We estimated a potential sample size of 1390 participants based on a 30% response rate from the estimate population of genetic and palliative health professionals, which would allow estimates of prevalence with a 95% CI half-width of <1%, and to test for differences in an indicator between subgroups (with at least 500 members) of 10% absolute difference with 0.9 power.

We began recruiting participants through professional organisations in March 2022 and closed the survey on the 21st July 2022. Three palliative care organisations (Australia and New Zealand Society of Palliative Medicine, Palliative Care Nurses Australia, and Palliative Care Nurses New Zealand) and one genetic organisation (Human Genetics Society of Australasia, including two of its special interest groups: Australasian Society of Genetic Counsellors and Australasian Association of Clinical Geneticists) advertised the survey link to their members via email blasts and online newsletters. Organisations circulated the invitation up to three times. Health professionals self-selected to participate. On the survey landing page, participants selected whether they were a palliative care or genetic health professional, and this directed them to the relevant survey based on their speciality.

### Instrument

We searched APA PsycTests for relevant validated scales [25]. We identified one scale that assessed hospice nurses’ perceptions of the importance of genetics to care and confidence performing ‘genetic-related activities’ [21]. However, this measure was not designed for genetic and non-genetic health professionals and was therefore not suitable. Instead, we developed two online surveys using REDCap software [26]; one for palliative care health professionals and the other for genetic health professionals (see supplementary file). The survey item development was informed by recent literature review findings [7, 13], our previous qualitative interviews and focus groups of genetic and palliative care health professionals (*n* = 40) [16, 17] and underpinned by the World Health Organisation Innovative Care for Chronic Conditions framework [27]. Across the two surveys, most items had the same or similar wording to enable comparison between the genetic and palliative care groups.

Participants were given a modified Likert scale (i.e., never, occasionally, sometimes, usually, or always) to indicate the frequency of performing genetic activities. Previous training and experience were assessed by selecting the most appropriate answer from a predefined list. For some items, response totals are greater than 100% because participants could select more than one option. Likert scales assessed confidence (1 = not all confident to 5 = confident) and attitudes (1 = strongly disagree to 5 = strongly agree). A list of previously identified barriers (*n* = 18), facilitators (*n* = 13), and resources or tools (*n* = 12) were provided. Participants were instructed to select up to three responses from each list as their ‘main challenges’ and ‘most helpful’ facilitators, resources, or tools. At the beginning of the survey, we defined DNA banking and testing as a clinical activity, rather than research. We were not able to distinguish for each question whether participants were responding hypothetically or from experience.

The survey was piloted with 19 participants (four palliative care and 15 genetic health professionals), of which four participated in a qualitative interview to provide feedback on the survey readability, acceptability, and usability. Participants wanted improved clarity about what was intended by the question ‘What is your ethnicity?’. In response, we replaced this with three additional questions related to country of birth, cultural background and language spoken at home [28]. We also incorporated their suggestion to include a ‘not applicable’ option to most questions.

### Data analysis

After closing the survey, we summarised categorical variables with numbers and percentages stratified by profession (i.e., palliative care health professional or genetic health professional). We compared professions’ demographic variables and responses to identical questions about requests for initiation of genetic testing, confidence with genetic activities and attitudes towards integrating genetics into palliative care. Comparisons were made using Fisher-Freeman-Halton Exact Test or Chi-Square Test (using the exact test where there was an expected cell count <5), with statistical significance set at *α* < 0.05. For questions that were not designed to be compared, results were described with summary statistics. Where there was item non-response, we used listwise-deletion to deal with missing data. For summary statistics and comparisons, we used SPSS version 28 [29]. For visualisations, we used R (version 4.1.3) [30] and ggPlot [31].

### Ethics

The University of Technology Sydney Human Research Ethics Office granted ethical approval for this study (ETH19-2408/21-5854). The survey landing page provided participants with information about the study (see supplementary file). Consent was implied by completion of the survey.

## Results

We received 80 responses, of which seven were blank. Emails containing the survey invitation were opened by a maximum of 1438 potential participants, equalling an approximate response rate of 6%. We were unable to collect reasons for non-participation from non-responders. Eighteen participants provided partially completed responses that we included in the analysis, therefore, the frequency counts vary between items.

### Demographics

Demographic data was provided by 75% (*n* = 55/73) partipants and are presented in Table 1. Most participants were palliative care health professionals (*n* = 44/73, 60%), female (*n* = 51/55, 93%), born in Australia (*n* = 37/55, 67%), working in the public sector (*n* = 43/55, 78%) and a metropolitan location (*n* = 44/55, 80%). There were no significant differences between palliative care and genetic health professionals’ gender (*p* = 0.624), age (*p* = 0.686), years since qualification (*p* = 0.74), years of experience in specialist area (*p* = 0.367), work sector (*p* = 0.316) or location (*p* = 0.113). More genetic health professionals held a master’s degree than palliative care health professionals (*p* = 0.001).

**Table 1:** Demographic results overall and stratified by health profession.

Demographic variable	Overall (*n* = 55)*	Genetic HP (*n* = 24)	Palliative care HP (*n* = 31)
	*n* (%)	*n* (%)	*n* (%)
Gender			
Female	51 (93)	23 (96)	28 (90)
Male	4 (7)	1 (4)	3 (10)
Non-binary	0 (0)	0 (0)	0 (0)
Prefer not to disclose	0 (0)	0 (0)	0 (0)
Age			
20–24	2 (4)	2 (8)	0 (0)
25–34	12 (22)	6 (25)	6 (19)
35–44	16 (29)	7 (29)	9 (29)
45–54	11 (20)	4 (17)	7 (23)
55–64	13 (24)	5 (21)	8 (26)
>65	1 (2)	0 (0)	1 (3)
Country of birth			
Australia	37 (67)	19 (79)	18 (58)
New Zealand	2 (4)	0 (0)	2 (6)
England	9 (16)	1 (4)	8 (26)
Scotland	2 (4)	1 (4)	1 (3)
Other	5 (9)	3 (13)	2 (6)
Country of work			
Australia	48 (87)	24 (100)	24 (77)
New Zealand	7 (13)	0 (0)	7 (23)
Cultural background/ethnicity			
None	2 (4)	1 (4)	1 (3)
Australian	38 (69)	19 (79)	19 (61)
New Zealand	2 (4)	0 (0)	2 (6)
English	6 (11)	0 (0)	6 (19)
Irish	3 (5)	1 (4)	2 (6)
Scottish	2 (4)	1 (4)	1 (3)
Chinese	3 (5)	0 (0)	3 (10)
Other or prefer not to say	10 (18)	7 (29)	3 (10)
Language at home			
English	54 (98)	23 (96)	31 (100)
Other	1 (2)	1 (4)	0 (0)
Profession			
Medical	20 (36)	4 (17)	16 (52)
Nursing	15 (27)	0 (0)	15 (48)
Genetic Counselling	20 (36)	20 (83)	0 (0)
Highest qualification			
PhD	4 (7)	2 (8)	2 (6)
Master’s degree	20 (36)	15 (63)	5 (16)
Bachelor’s degree	19 (36)	3 (13)	16 (52)
Diploma	6 (11)	1 (4)	5 (16)
Professional qualification	6 (11)	3 (13)	3 (10)
Years since qualification			
Less than 2 years	5 (9)	4 (17)	1 (3)
2–5 years	3 (5)	2 (8)	1 (3)
6–10 years	13 (24)	5 (21)	8 (26)
11–15 years	11 (20)	7 (29)	4 (13)
More than 15 years	23 (42)	6 (25)	17 (55)
Years in specialist area			
Less than 2 years	9 (16)	5 (21)	4 (13)
2–5 years	9 (16)	3 (13)	6 (19)
6–10 years	12 (22)	3 (13)	9 (29)
11–15 years	11 (20)	7 (29)	4 (13)
More than 15 years	14 (25)	6 (25)	8 (26)
Work sector			
Public	43 (78)	18 (75)	25 (81)
Private	3 (5)	2 (8)	1 (3)
Public and private	7 (13)	2 (8)	5 (16)
Other	2 (4)	2 (8)	0 (0)
Work location			
City/Metro/Urban	44 (80)	22 (92)	22 (71)
Regional	8 (15)	1 (4)	7 (23)
Rural	2 (4)	1 (4)	1 (3)
Other	1 (2)	0 (0)	1 (3)
Work setting			
Hospital	34 (62)	21 (88)	13 (42)
Hospice	9 (16)	0 (0)	9 (29)
Community clinic	4 (7)	0 (0)	4 (13)
Home care	3 (6)	0 (0)	3 (10)
Independent clinic	3 (6)	1 (4)	2 (6)
Other	2 (4)	2 (8)	0 (0)

*Demographic information was missing from 18 surveys, therefore, *n* = 55.

### Previous experience in genetics and palliative care

Palliative care health professionals indicated that they perform the following three activities at least ‘occasionally’: taking a family health history (*n* = 34/43, 79%), drawing a three-generation pedigree (*n* = 22/43, 51%) and making a genetic risk assessment (*n* = 15/43, 35%). The most common time to take a family health history was when the patient commenced palliative care (*n* = 21/43, 49%). Half of the palliative care health professionals (*n* = 22/42, 52%) indicated that in the previous year, they had not checked if their patient, or their relatives, had already had an opportunity to discuss genetics before coming under their care.

Most genetic and palliative care health professionals indicated requests for genetic testing come from oncology health professionals (*n* = 21/55, 38%), followed by family members (*n* = 14/55, 25%). Only 2% (*n* = 1/55*)* indicated that requests for genetic testing come from palliative care health professionals. Many genetic health professionals had been involved with facilitating DNA banking or testing for a person receiving palliative care (*n* = 23/28, 82%). Among these participants, the most frequently selected time that they became involved was when the patient was close to death (*n* = 11/23, 48%).

### Previous training in genetics and palliative care

Almost all palliative care health professionals had not received previous training in genetic risk assessment or testing (*n* = 40/44, 91%) but the majority (*n* = 30/40, 75%) were interested in receiving training. For those who were not interested (*n* = 10/40, 25%), reasons included having other educational priorities (*n* = 3/10, 33%), genetics not being relevant to their work (*n* = 3/10, 33%), lack of time (*n* = 2/10, 20%) or being retired or close to retirement (*n* = 2/10, 20%). More than half of the genetic health professionals had previously received training in communicating with patients at end of life or bereaved families (*n* = 17/29, 59%). All genetic health professionals without previous training were interested in receiving training (*n* = 12/12, 100%).

### Confidence integrating genetics into palliative care

A third of palliative care health professionals were ‘fairly confident’ or ‘confident’ with contacting their local genetics service (*n* = 11/33, 33%) and a quarter were ‘fairly confident’ or ‘confident’ assessing when to broach a genetics discussion (*n* = 8/33, 24%). Fewer palliative care health professionals were ‘fairly confident’ or ‘confident’ identifying patients who were eligible for genetic testing (*n* = 4/33, 12%) and responding to family members’ questions about genetics (*n* = 4/33, 12%). Most genetic health professionals were ‘fairly confident’ or ‘confident’ when communicating with patients (*n* = 15/25, 60%) and families (*n* = 20/25, 80%) at end of life.

**Fig. 1:**
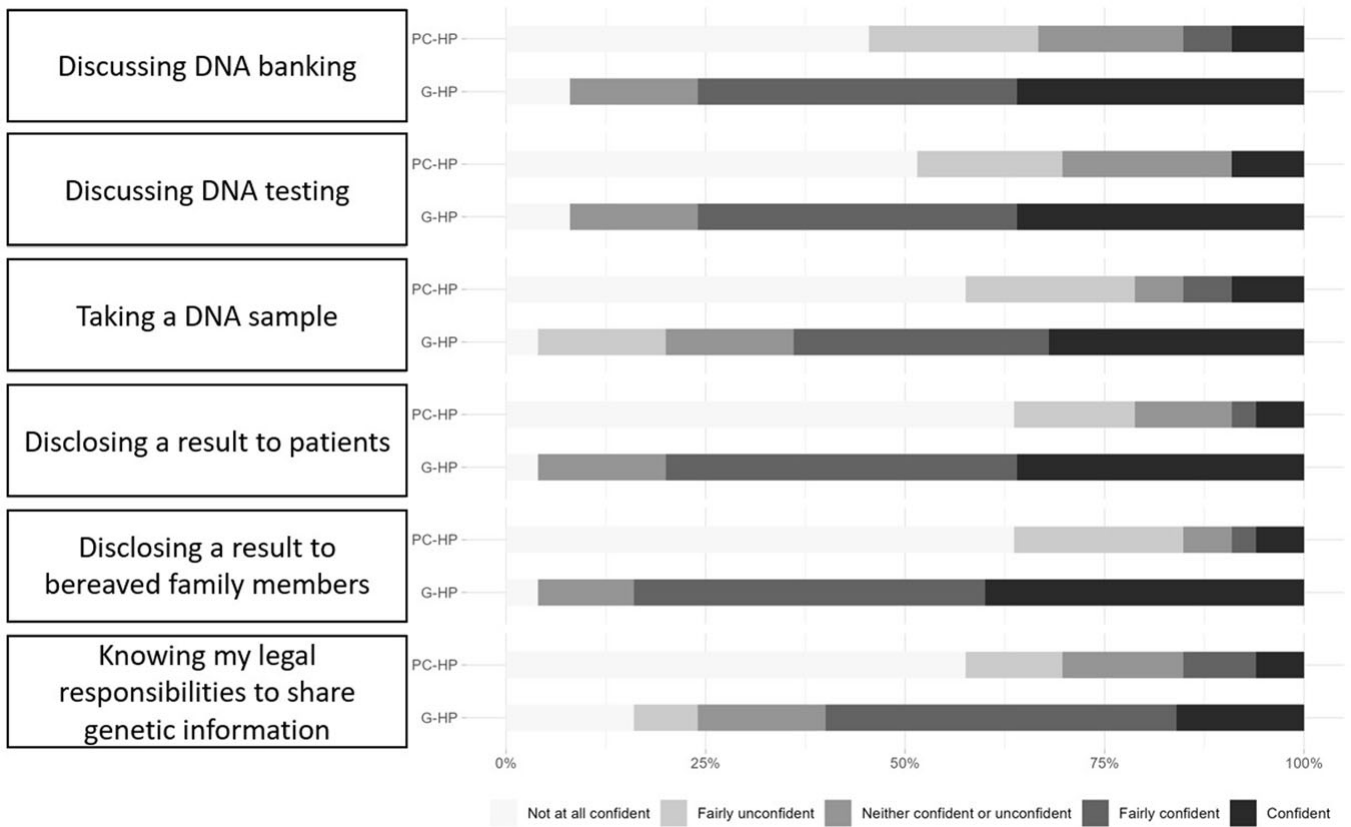
Confidence with genetic activities. Palliative care (PC-HP, *n* = 33) and genetic health professionals (G-HP, *n* = 25) rate their confidence in engaging with six different genetic activities.

When comparing the two health professional groups (Fig. 1), palliative care health professionals reported lower confidence than genetic health professionals when discussing DNA banking (*p* = 0.001) or testing (*p* = 0.001), facilitating or taking a DNA sample (*p* = 0.001), disclosing genetic results to palliative patients (*p* = 0.001) or bereaved family members (*p* = 0.001), and navigating legal responsibilities when sharing genetic information in the palliative context (*p* = 0.003).

### Perceived barriers and facilitators

The most frequently selected barrier by genetic and palliative care health professionals was palliative care health professionals’ lack of knowledge about DNA banking/testing (Table 2; *n* = 32/72, 44%). Genetic health professionals selected the under-referral of palliative patients to genetic services as a barrier more frequently than palliative care health professionals (*p* = 0.001). Palliative care health professionals selected ‘identifying eligible patients’ as a barrier more frequently than genetic health professionals (*p* = 0.046).

**Table 2:** Participants selected their top three perceived barriers and facilitators, and resources or tools they had found useful.

Item description	Overall (*n* = 72)	G-HP (*n* = 29)	PC-HP (*n* = 43)
	*n* (%)	*n* (%)	*n* (%)
Top 5 barriers			
PC-HPs’ lack of knowledge	32 (44)	13 (45)	19 (44)
Identifying eligible patients	19 (26)	4 (14)	15 (35)
Conflicting priorities between providing palliative care and genetic testing	15 (21)	6 (21)	9 (21)
Under-referral of palliative patients to genetics	15 (21)	12 (41)	3 (7)
Urgency of the situation/referral	13 (18)	8 (28)	5 (12)
Top 5 facilitators			
Developing a specific genetic referral template for palliative care patients	31 (43)	9 (31)	22 (51)
Fostering closer working relationships between PC-HPs & G-HPs	27 (38)	15 (52)	12 (28)
G-HPs deliver education to PC-HPs	25 (35)	11 (38)	14 (33)
Embedding a genetic counsellor in the palliative care team	17 (24)	8 (28)	9 (21)
PC-HPs & G-HPs attend the same multidisciplinary team meetings	15 (21)	11 (38)	4 (9)
Top 5 resources or tools			
Support from a specialist genetics service or colleague	33 (46)	19 (66)	14 (33)
Support from a palliative care colleague	15 (21)	9 (31)	6 (14)
I have not found any resources or tools helpful	10 (14)	3 (10)	7 (16)
Other/no experience	10 (14)	1 (3)	9 (21)
Clinical decision-making algorithm or guideline	9 (13)	3 (10)	6 (14)

Items are ranked in order of the most frequently selected across both genetic (G-HP) and palliative care health professional (PC-HP) groups.

Total % exceeds 100% as participants could select more than one option. The full lists of barriers, facilitators & resources or tools are in the supplementary file.

Genetic health professionals considered fostering closer working relationships between palliative care health professionals and genetic health professionals a more important facilitator than palliative care health professionals (*p* = 0.041). Palliative care and genetic health professionals frequently selected the development of a specific referral template as a facilitator (*p* = 0.145).

Of nine resources or tools to support palliative-genetic DNA banking or testing, the most frequently selected by both groups was ‘support from a specialist genetics service or colleague’ (*n* = 33/72, 46%), although this was more frequently selected by genetic health professionals (*p* = 0.006).

### Attitudes towards genetics in palliative care

**Fig. 2:**
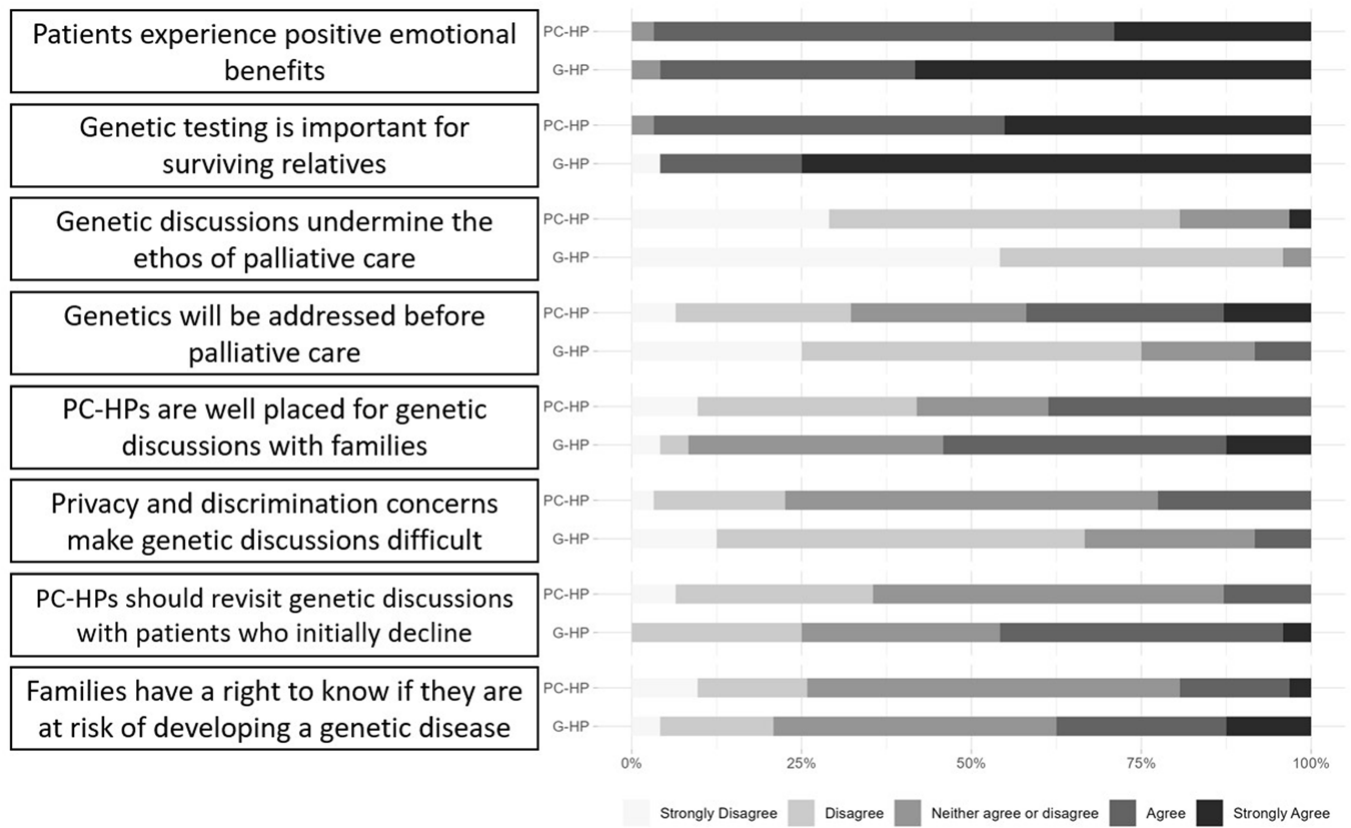
Attitudes towards integrating genetics into palliative care. Palliative care (PC-HP, *n* = 31) and genetic health professionals (G-HP, *n* = 24) rated their agreement with statements related to the integration of genetics into palliative care.

Nearly all the genetic (*n* = 23/24, 96%) and palliative care health professionals (*n* = 30/31, 97%) ‘agreed’ or ‘strongly agreed’ that palliative patients may experience positive emotional benefits from genetic counselling or testing (*p* = 1.0; Fig. 2). The majority of genetic (*n* = 23/24, %) and palliative care health professionals (*n* = 30/31, %) ‘agreed’ or ‘strongly agreed’ that genetic testing may be important for surviving family members (*p* = 0.687). Most genetic (*n* = 23/24, 96%) and palliative care health professionals (*n* = 25/31, 81%) ‘strongly disagreed’ or ‘disagreed’ that discussing DNA banking/testing with people receiving palliative care undermines the central ethos of palliative care (*p* = 0.286). More genetic health professionals disagreed (*n* = 12/24, 50%) that DNA banking/testing will have been discussed prior to palliative care than palliative care health professionals (*n* = 8/31, 26%, *p* = 0.018). Palliative care health professionals (*n* = 10/31, 32%) disagreed more frequently than genetic health professionals (*n* = 1/24, 4%) that ‘palliative care health professionals are well placed to have discussions about DNA banking/testing with family members’ (*p* = 0.014). Genetic health professionals more frequently disagreed (*n* = 13/24, 54%) that privacy and discrimination concerns make DNA banking/testing discussions difficult compared to palliative care health professionals (*n* = 6/31, 19%; *p* = 0.01). More genetic health professionals agreed (*n* = 10/24, 42%) that palliative care health professionals should revisit genetics discussions with palliative patients if they initially decline compared to palliative care health professionals (*n* = 4/31, 13%; *p* = 0.039). For the statement ‘The family of a palliative patient have a right to know if they are at risk of developing a genetic disease, regardless of the palliative patient’s wishes,’ the majority of genetic (*n* = 10/24, 42%) and palliative care health professionals (*n* = 17/31, 55%) neither agreed nor disagreed (*p* = 0.562).

## Discussion

This survey found that genetic and palliative care health professionals are supportive of integrating genetics into the care of people with palliative care needs and their families, although some differences in opinion regarding the role of palliative care health professionals were noted. We identified knowledge and confidence barriers along with intervention targets, including relationship building between genetic and palliative care health professionals, and potential improvements to referral processes.

Palliative care health professionals reported low levels of confidence when engaging with genetic activities, consistent with previous reports [4, 14, 15]. Our results further current understanding about palliative care health professionals’ lack of confidence by identifying potential areas where support may improve capability. For example, a targeted educational approach that focuses on broaching DNA banking and testing discussions, facilitating DNA collection, and understanding the legalities of sharing genetic health information may be of greater support to palliative care health professionals than delivering general genetics education.

Despite their low confidence, most palliative care health professionals would at least occasionally obtain a family health history from their patient. Less frequently, palliative care health professionals were drawing a three-generation pedigree or conducting a genetic risk assessment. One way to support palliative care health professionals’ engagement and confidence in pedigree drawing as the basis of a genetic risk assessment may be to leverage their existing skill and knowledge about genograms. Within palliative medicine, genograms are often used to document family structures, relationships, and other social information [32]. Genetic health professionals’ expertise makes them well placed to deliver education about pedigree-drawing and risk assessment to palliative care health professionals. In keeping with previous efforts to upskill non-genetics health professionals, our findings indicated that education delivered by genetic health professionals would be highly valued [33].

Genetic health professionals indicated that patients with palliative care needs were under-referred to genetic services. At least two audits of referrals to genetic services reported related findings. One audit found that 22% of unaffected relatives referred for risk assessment were received 11 years (on average) after the last affected individual had died [34]. A second audit investigated the referrals of 45 individuals who died while awaiting a genetics appointment. They estimated the health of 133 first-degree relatives was moderately or significantly impacted by their family member failing to receive a genetics appointment [35]. These suboptimal practices may explain why genetic health professionals in our cohort were less likely to think that genetics will have been addressed prior to patients receiving palliative care (i.e., by treating clinicians, such as oncologists) compared to palliative care health professionals.

Another possible explanation, given the close links between palliative care and oncology, is the impact of cancer mainstreaming models upon palliative care health professionals’ assumptions about genetic referral practices. For example, it is now common in Australia for gynaecological oncologists to organise germline breast and ovarian cancer gene testing rather than referring the patient to a genetic service [33]. Palliative care health professionals may therefore assume that all genetic needs will be addressed prior to referral to palliative care, and not consider it to be their responsibility. However, mainstreaming models only target patients with distinct tumour types (e.g., high-grade serous ovarian carcinoma) and there is a lack of mainstreaming in other specialties, such as neurology or cardiology [36]. Furthermore, research has shown that, even when cancer patients receive germline results through mainstreamed testing, oncologists report low confidence explaining implications to family members [37]. If this is the case, we suggest palliative care health professionals check all palliative care patients’ genetic needs, particularly for those with ‘non-mainstreamed’ malignancies or a non-malignant disease. However, our findings suggest palliative care health professionals are not routinely verifying this information.

Our results raise questions about genetic and palliative care health professionals’ views on the palliative care role in addressing genetics. In keeping with recent reports, most palliative care health professional participants agreed they had some responsibility to address genetics with their patients, although less certainty was evident when considering their responsibility to family members [16]. If, as our findings show, family members are often initiating genetic discussions, a better understanding of how palliative care health professionals respond to family members queries is needed. As family-centred care is a central tenet of palliative care, it seems appropriate for palliative care health professionals to address family members’ genetic concerns or refer them to an appropriate provider for more complex discussions [38]. In contrast, genetic health professionals agreed that palliative care health professionals were well placed for discussions about genetics with family members. We suggest there may be a mismatch between what genetic health professionals expect of palliative care health professionals and what is happening in practice. Incorporating communication about genetics to family members may therefore also be an important topic to include in an educational intervention. Future research to understand palliative care health professionals’ views and experiences of communicating about genetics with family members, as opposed to patients, would provide a valuable insight into the content of these discussions, reasons for discomfort and avenues for support.

Our results did not confirm previous concerns from palliative care health professionals about the potential harms of addressing genetics, such as a negative psychological impact to patients and relatives [39]. The benefits of genetic information for palliative patients and their families were almost unanimous. Participants rejected the idea that the palliative care context is an ‘inappropriate’ place to discuss genetics, which contrasts with previous qualitative work [16, 20]. Although we do not discount these potential harms, our results may reflect a shift in attitudes as genetics in routine medical care becomes more widely accepted [40].

While a referral template appears to be an important facilitator, genetic health professionals were also interested in interventions that preceded the point of referral. The facilitators supported by genetic health professionals are similar to interventions implemented in various mainstreaming models, including fostering collaborative relationships, embedding a genetic counsellor in the palliative care team, and multidisciplinary team meetings [41, 42]. It is possible genetic health professionals were more likely to emphasise these ‘collaborative’ interventions because of a belief that genetics is not valued by palliative care health professionals [17]. Genetic health professionals may view collaborative working as an opportunity to educate palliative care health professionals about the familial benefits of genetic counselling and testing, in addition to facilitating referrals. Interestingly, our findings do not support the suggestion that palliative care health professionals do not value genetic counselling and testing. Rather, palliative care health professionals simply desire practical and educational support.

### Strengths and limitations

Although several advertisements were sent through national organisations, our sample was small and self-selected, so may be subject to non-response/selection bias. The validity of comparisons would have been improved with larger cohorts. Our findings may not be generalisable (that is, the data presented here may not represent their source groups) or represent diverse attitudes towards integrating genetics into palliative care. Further work to understand reasons for the low response rate could provide valuable insights, including whether the topic was perceived as unimportant or if it were a result of survey fatigue (several participants dropped out halfway through the survey).

Our ability to conduct the planned statistical analysis, including ordinal logistic regression for Likert responses, was also impacted by the small sample size. Furthermore, though participants reported their engagement with genetic activities, such as taking a family history, we were unable to determine the quality or content of these activities.

The professional organisations who circulated invitations to the members for participation were only able to share limited demographic summaries. For example, one organisation could only share the number of members who were qualified or in-training. As a result, we could not reliably assess the representativeness of our sample. Genetic health professionals were more likely to hold a Master degree than palliative care health professionals. This could be explained by the proportion of genetic counsellors in the sample, for whom a Master degree has been the entry level qualification since 2010 [43].

Despite these limitations, our evidence begins to fill the thematic and methodological literature gaps in this understudied area. Further work with patients who have palliative care needs, and their families, would be likely to identify additional barriers and facilitators to help us understand and support the integration of genetics into their care.

## Conclusion

Genetic and palliative care health professionals both support the integration of genetics into the routine care of people with palliative care needs and their families. Building the confidence of palliative care health professionals through the delivery of education by genetic health professionals, inter-specialty collaboration, and development of a specific genetic referral template is an important first step. Defining the role of palliative care health professionals in addressing genetics with family members requires further work. Our findings shine a light on the existing barriers and facilitators to integrate genetics into the care of people with palliative care needs with a view to developing targeted interventions. In doing so, the benefits of genetic counselling and testing can be realised by patients with palliative care needs and their families.

### Supplementary information

Supplementary material

## Data Availability

Data pertaining to this study is freely available on the Open Science Framework: https://tinyurl.com/4exmv6hm.
