# Supplementary material for: A survey of genetic and palliative care health professionals’ views of integrating genetics into palliative care
Source: Eur J Hum Genet. 2023 Jun 21;32(1):109–16. doi: 10.1038/s41431-023-01409-6 (PMC10772073; doi:10.1038/s41431-023-01409-6)
Supplement: Supplementary file 1 — Supplementary material [file 41431_2023_1409_MOESM1_ESM.docx]

***A survey of genetic & palliative care health professionals’ views of integrating genetics into palliative care***

Stephanie White^1^, Dr Erin Turbitt^1^, Associate Professor Kris Rogers^1^, Associate Professor Kathy Tucker^2,3^, Associate Professor Alison McEwen^1^, Associate Professor Megan Best^4^, Professor Jane L. Phillips^4^, Dr Chris Jacobs^1^

**Affiliations**

^1^ Graduate School of Health, University of Technology Sydney, NSW, Australia

^2^ Hereditary Cancer Centre, Nelune Comprehensive Cancer Centre, Prince of Wales Hospital, NSW, Australia

^3^ Prince of Wales Clinical School, Division of Medicine and Health, University of New South Wales, NSW, Australia

^4^ Institute for Ethics and Society, University of Notre Dame Australia, NSW, Australia

^5^ School of Nursing, Faculty of Health, Queensland University of Technology, QLD, Australia

**SUPPLEMENTARY FILE**

Contents

[Appendix A: Survey for Genetics Health Professionals 1](#_Toc120890925)

[Appendix B: Survey for Palliative Care Health Professionals 16](#_Toc120890926)

[Appendix C: Participant Information Sheet (Survey landing page) 31](#_Toc120890927)

[Appendix D: Full list of barriers, facilitators, and resources/tools 33](#_Toc120890928)

# Appendix A: Survey for Genetics Health Professionals

1. Are you now working, or have you previously worked, in Yes a clinical area? No
2. Which organisation sent you the link to the survey Human Genetics Society of Australasia (HGSA) that you are completing? Australasian Society of Genetic Counsellors (ASGC)

Australasian Association of Clinical Geneticists (AACG)


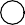

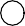

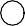

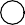

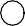


British Society for Genetic Medicine (BSGM) Association of Genetic Nurses and Counsellors (AGNC)


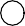

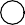


Clinical Genetics Society (CGS)


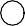

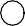

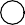


European Society of Human Genetics (ESHG) Other

Please explain.

The following questions are to understand your experiences and views about discussing DNA banking/testing with patients receiving palliative care, and their families.

We will ask you some demographic questions at the end of the survey. Please note:

DNA banking, as opposed to DNA testing, is the process of obtaining a DNA sample (usually blood, saliva or buccal) and storing this sample in a laboratory, without performing any DNA testing. As you answer these questions, please consider DNA banking for CLINICAL use only. This means the DNA would be used for future DNA testing to help understand genetic risk for relatives and NOT used for research purposes.


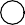

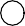


1. Have you ever received any training in communicating Yes with patients at the end of life or with bereaved No families?

What area(s) did you study? (Please indicate all that are relevant.)

Communicating with cancer patients Communication skills with patients at the end of life

Bereavement counselling Other

Please explain.

What type of training have you received in communicating with patients at the end of life or with bereaved families? (Please indicate all that are relevant.)

Degree/diploma

Short course/module over at least two sessions One-off lecture/seminar/workshop

On-line short course or Massive Online Open Course (MOOC)

Course/session on communication with people receiving palliative care as part of another course or study day

Course/session on bereavement counselling Private study (e.g. reading papers)

Other

Please explain.

How long has it been since you last received training Less than 12 months or education in communicating with patients at the end 1 to 2 years

of life or with bereaved families? 3 to 5 years 6 to 10 years


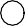

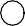

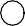

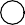

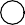


More than 10 years

Are you interested in receiving training in Yes

communicating with patients at the end of life or with No bereaved families?


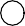

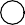


What type of training would you like to receive in communicating with patients at the end of life or with bereaved families? (Please indicate all that are relevant.)

Degree/diploma

Short course/module over at least two sessions One-off lecture/seminar/workshop

On-line short course or Massive Online Open Course (MOOC)

Course/session on communication with people receiving palliative care as part of another course or study day

Course/session on bereavement counselling Private study (e.g. reading papers)

Other

Please specify

What is the main reason you are not interested in Lack of time to receive training receiving this training? I have other education/training priorities

I already know a lot about communicating with patients at end-of-life & bereaved families


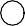

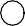

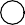


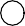
 Communicating with patients at end-of-life & bereaved families is not relevant to my work


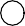
 Other

Please specify

Please add any comments about the training you have received or would find helpful in communicating with patients at the end of life of with bereaved families.

1. Have you ever been involved in facilitating DNA Yes banking/testing for people receiving palliative care? No

Not sure


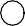

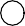

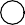


4a. In your experience, at what point did you usually When the patient commences palliative care become involved? When the patient is close to death

After the patient has died


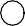

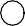

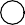

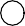

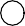

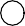


It has never been raised in my experience Not sure

Other

Please explain.

1. In your experience, who usually initiates requests for Patients

DNA testing? Family members


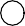

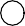

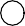

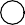

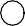

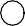

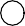

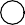


Palliative care health professionals Genetics health professionals Oncology health professionals

It has never been raised in my experience Not sure

Other

Please explain.

6a. Please indicate approximately how often you have been involved in the following activities in the last 12 months.

Zero Once or twice Three to five

times

Six to 10 times

More than 10 times

I have not worked clinically in the last 12 months

1. Identifying a patient receiving
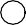

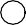

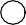

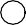

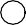

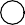
 palliative care who is eligible for

DNA banking/testing.

1. Initiating a discussion about DNA
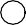

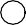

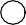

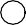

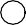

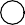
 banking/testing with a patient

receiving palliative care or their relative.

1. Providing advice to a palliative
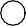

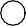

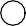

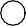

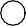

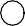
 care health professional about

one of their patients

d Receiving a referral from a
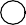

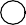

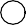

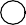

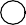

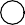
 palliative care health

professional for a patient receiving palliative care

1. Taking consent for DNA
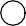

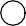

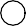

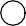

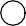

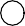
 banking/testing from a patient

receiving palliative care.

1. Taking consent for DNA
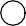

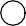

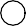

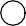

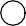

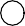
 banking/testing from a relative

of a patient receiving palliative care.

6b. Please indicate approximately how often you have been involved in the following activities in the last 12 months.

Zero Once or twice Three to five

times

Six to 10

times

More than 10

times

I have not

worked clinically in the last 12 months

1. Facilitating collection of a DNA
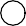

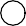

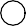

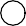

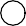

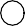
 sample from a patient receiving

palliative care

1. Disclosing genetic/genomic test
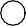

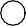

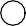

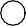

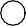

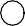
 results to a patient receiving

palliative care.

1. Disclosing a deceased patient's
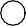

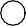

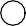

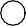

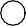

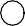
 genetic test results to bereaved

relatives.

1. Providing genetic counselling to
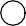

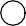

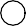

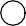

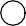
 a patient receiving palliative

care

1. Which scenario best describes the availability of Specialist genetics services and palliative care specialist genetics services for people receiving services are embedded within the same hospital or palliative care in your area? group of hospitals.

Specialist genetics services and palliative care services are not embedded within the hospital or group but are accessible to each other.

Specialist genetics services and palliative care services are NOT accessible to each other.

Palliative care services have access to private genetics services only

Not sure

1. In your experience how is consent for DNA banking or Verbal consent only (undocumented) testing obtained and documented? Verbal consent (documented in the patient's

clinical records)

Written consent using locally available paperwork (but not a formal genetics consent form)

Written consent using a formal genetics consent form

In my experience consent has not been taken Other

Please explain.

1. In your experience, what do you consider to be the main challenges for genetics health professionals in facilitating DNA storage/testing for people receiving palliative care? (Please select up to 3 responses.)

* Please note: These response options have all been identified as challenges in prior studies. For the purpose of identifying priority areas, we are requesting you nominate your top three challenges

Identifying eligible patients Urgency of the situation/ referral

Conflicting priorities between providing palliative care and facilitating genetic testing Obtaining informed consent

Discomfort with initiating discussions about DNA storage/testing with patients or families in palliative care

Palliative care health professionals' lack of knowledge about DNA banking/testing or procedures Genetics health professionals' lack of knowledge

of the procedure for consent and DNA storage The views or expectations of the family

Lack of availability of specialist genetics services

Communication difficulties between genetics and palliative care services

Conflicting views within the palliative care team about the utility of DNA banking/testing for palliative patients, and their families

Lack of resources

Distress of the patient or family members Complex family dynamics

Concern that a discussion about genetics could damage the therapeutic relationship

Under-referral of palliative patients to genetics services

In my experience DNA storage or genetic/ genomic testing has not ever been considered

Other

(Please choose only up to 3 responses)

1. Please indicate your level of confidence about the following:

Not at all

confident

Fairly

unconfident

Neither confident

or unconfident

Fairly confident Confident

- 1. Communicating with patients at the end of life
  2. Communicating with the families of patients who are at the end of

life

- 1. Discussing DNA banking with patients or their families in the

palliative care setting

- 1. Discussing DNA testing with patients or their families in the

palliative care setting

- 1. Facilitating collection of a DNA sample from a patient receiving

palliative care

- 1. Disclosing genetic/genomic test results to palliative care patients
  2. Disclosing genetic/genomic test results to bereaved families
  3. Knowing my legal responsibilities when sharing

health information with family members when a patient is terminal or after they have died

1. What resources or tools have you found helpful when communicating with patients receiving palliative care and/or their families? (Please indicate all that are relevant.)

Web-based risk assessment tool Smart phone App

Support from a palliative care colleague Support from a genetics colleague Educational brochures

Telephone information hotline Face to face education

Online education

Clinical decision-making algorithm Clinical practice guidelines

I have not found any resources or tools helpful Other

How frequently did you receive "face to face

education" for communicating with patients receiving palliative care and/or their families?

Which clinical practice guidelines did you find useful?

1. What additional resources or tools would be helpful, if any?
2. Please indicate to what extent you agree or disagree with the following statements:
   1. Discussing DNA banking/testing with people receiving palliative care undermines the central ethos of palliative care in providing comfort and support at an emotionally vulnerable time.

Strongly

disagree

Disagree Neither agree

nor disagree

Agree Strongly agree

- 1. Patients may experience positive emotional benefits from being

able to give a sample for DNA banking/testing for the possible future benefit of their relatives.

- 1. Discussing DNA banking/testing may cause distress to the

families by making them assume/fear their fate is pre-determined.

- 1. DNA banking/testing will have been discussed by other health

professionals before the patient is referred to palliative care.

- 1. The priority in palliative care is to improve quality of life and

relieve suffering and therefore it is not an appropriate time to discuss DNA banking/testing.

- 1. DNA banking/testing of the patient may be important for the

surviving relatives.

- 1. It is not the responsibility of health professionals in palliative

care to discuss DNA banking/testing.

- 1. DNA banking/testing is not appropriate for people receiving

palliative care because it will not help the patient.

1. Please indicate to what extent you agree or disagree with the following statements:
   1. Palliative care health professionals are well placed to have discussions about DNA banking/testing with the family members of a palliative patient

Strongly disagree

Disagree Neither agree nor disagree

Agree Strongly agree

- 1. Concerns about genetic discrimination (eg. insurance

and employment discrimination) make discussions about DNA banking/testing with palliative patients and their families difficult

- 1. Families generally appreciate being told genetic information

that is relevant to their health

- 1. Discussions about DNA banking/testing need to be

individualised to the palliative patient, and their families

- 1. As time goes on, genetic health information will inevitably

become part of the palliative care health professionals' scope of practice

- 1. If a patient declines a discussion about DNA banking/testing ,

palliative care health professionals should revisit this discussion with palliative patients at a later date to see if they've changed their mind

- 1. The family of a palliative patient have a right to know if they are

at risk of developing a genetic disease, regardless of the palliative patient's wishes

- 1. The right of a palliative patient to decline a discussion about

DNA banking/testing are to be respected, regardless of the family's wishes

1. In your opinion, which of these would most help genetics health professionals to discuss and/or facilitate DNA banking/testing with palliative patients, and their families? (Please choose up to 3 responses)

*Please note: These response options have all been identified as facilitators in prior studies. For the purpose of identifying priority areas, we are requesting you nominate your top three facilitators

Physically co-locating palliative care and genetics health professionals within a hospital or organisation

Developing a specific referral template for palliative care patients to the genetics service that includes relevant family member details Embedding a genetic counsellor in the palliative care team

Having both palliative care and genetics health professionals attending the same multidisciplinary team meetings

Fostering a closer working relationships between palliative care and genetics health professionals Delivering genetics education to palliative care health professionals, including ways of sensitively communicating with patients and families about genetics

Policy guidance detailing how and when to discuss DNA banking/testing with palliative patients and their families

Empowering palliative patients, and their families, to seek out DNA banking/testing for themselves

Speaking directly to the palliative care health professional about the palliative patient Improving the capability of electronic medical records to share relevant information between health professionals

Collaborating with palliative care health professionals to facilitate collection of a DNA sample from a palliative patient

We shouldn't be discussing DNA banking/testing with palliative patients, or their families

Other

Please explain

1. Previous research suggests that patients who are suitable for DNA testing and close to end of life should be offered DNA banking by palliative care health professionals, rather than referring them to the genetics service for DNA testing.

We would like to understand your views about how this would work in practice.

Please indicate your comfort with palliative care health professionals performing the following actions:

- 1. Introduce the idea of DNA banking with the patient and/or the family

Very

uncomfortable

Somewhat

uncomfortable

Neutral Somewhat

comfortable

Very comfortable

- 1. Obtain consent for DNA banking from the patient, or appointed

representative

Facilitate collection of DNA sample

- 1. Organise for the DNA sample to be banked
  2. Instruct the family how to follow up with the genetics service
  3. Communicate family follow up plan to the genetics service

Please describe any further thoughts you have about palliative care health professionals performing these actions

**Thank you. We will now ask you some demographic questions.**

1. What is your gender? Female

Male

Self-described (specify below) Prefer not to say

Please describe

1. What is your age group? < 20

20-24

25-34

35-44

45-54

55-64

65 or older Prefer to not say

1. What is your country of birth? Australia New Zealand England India Philippines Vietnam Italy

South Africa Malaysia Scotland

Other (please specify) Prefer not to say

Please specify

1. What cultural background or ethnicity do you identify with? (Select all that apply)

None Australian New Zealand English

Irish Scottish Chinese Italian Indian German Greek Vietnamese

Other (please specify)

Prefer not to say

(You may select more than one)

Please describe your cultural background or ethnicity

1. Which language do you mainly speak at home? (If more English than one language, indicate the one that is spoken Mandarin

most often) Arabic

Cantonese Vietnamese Italian Greek

Hindi Spanish Punjabi

Other (please specify) Prefer not to say

Please describe the language you mostly use at home

1. What is your primary profession? Medical Nursing

Genetic counselling Other

Please explain.

1. How many years has it been since you qualified in your Less than 2 years current profession? 2 to 5 years

6 to 10 years

11 to 15 years More than 15 years

1. What is your highest academic achievement? PhD

Master degree Bachelor degree Diploma

Professional qualification Other

Please explain.

1. What is your primary area of specialty? Clinical genetics Cancer genetics Other

Please explain.

1. What is your job title? Genetic Counsellor (not yet Registered/Certified) Genetic Counsellor (Member of the HGSA) Genetic Counsellor (Fellow of the HGSA)

Genetic Counsellor (GCRB Registered) Registered nurse

Senior registered nurse (including nurse practitioner)

Junior doctor (eg intern, resident or equivalent) Senior doctor (eg registrar, fellow or equivalent) Consultant doctor (including staff specialist or equivalent)

Other

Please explain.

1. How long have you worked in your current specialist Less than 2 years area? 2 to 5 years

6 to 10 years

11 to 15 years More than 15 years

1. Which country do you work in? Australia New Zealand England Scotland

Northern Ireland Wales

Other

Please explain.

1. Which sector do you usually work in? Public Private

Public and private Other

Please explain.

1. What location do you usually work in? City/metropolitan/urban Regional

Rural Other

Please explain.

1. What setting do you usually work in? Hospital Independent clinic Other

Please explain.

**Many thanks for your participation in this survey.**

**If you have any further comments, you may write them below. When you are finished, please press submit.**

**Have a great day!**

1. Please add any further comments about facilitating DNA banking/testing with people receiving palliative care

or their families.

1. Please tell us if you have any further comments about this survey or study.

# Appendix B: Survey for Palliative Care Health Professionals

1. Are you now working, or have you previously worked, in Yes a clinical area? No
2. Which organisation sent you the link to the survey Australian and New Zealand Society for Palliative that you are completing? Medicine (ANZSPM)

Palliative Care Nurses Australia (PCNA) Palliative Care Nurses New Zealand (PCNNZ) Association for Palliative Medicine (APM) Royal College of Nursing (RCN)

Palliative Care Forum Other

Please explain.

The following questions are to understand your experiences and views about discussing DNA banking/testing with patients receiving palliative care, and their families.

We will ask you some demographic questions at the end of the survey. Please note:

DNA banking, as opposed to DNA testing, is the process of obtaining a DNA sample (usually blood, saliva or buccal) and storing this sample in a laboratory, without performing any DNA testing As you answer these questions, please consider DNA banking for CLINICAL use only This means the DNA would be used for future DNA testing to help understand genetic risk for relatives and NOT used for research purposes.

1. Have you ever received any training in family history Yes risk assessment, genetic testing and/or genomic No testing?

What area(s) did you study? (Please indicate all that are relevant)

Family history risk assessment Genetics and/or genetic testing Genomics and/or genomic testing Other

Please explain.

What type of training have you received in family history risk assessment, genetic testing and/or genomic testing? (Please indicate all that are relevant)

Degree/diploma

Short course/module over at least two sessions One-off lecture/seminar/workshop

On-line short course or Massive Online Open Course (MOOC)

Courses/sessions on genetics/genomics as part of another course or study day

Private study (e.g. reading papers) Other

Please explain.

How long has it been since you last received training Less than 12 months or education in family history risk assessment, 1 to 2 years

genetic testing and/or genomic testing? 3 to 5 years 6 to 10 years

More than 10 years

Are you interested in receiving training in family Yes

history risk assessment and genetic/genomic testing? No

What type of training would you like to receive in family history risk assessment, genetic testing and/or genomic testing? (Please indicate all that are relevant)

Degree/diploma

Short course/module over at least two sessions One-off lecture/seminar/workshop

On-line short course or Massive Online Open Course (MOOC)

Courses/sessions on genetics/genomics as part of another course or study day

Private study (e.g. reading papers) Other

Please explain

What is the main reason you are not interested in Lack of time to receive training receiving training? I have other education/training priorities

I already know a lot about genetics/genomics Genetics/genomics is not relevant to my work Other

Please explain

Please add any comments about the training you have received or would find helpful about genetics/genomics.

1. Please indicate how often you are involved in the following activities in your current practice.

Never Occasionally Sometimes Usually Always

- 1. Taking a family health history
  2. Drawing a three-generation family tree (pedigree)
  3. Making a genetic risk assessment

1. In your experience, at what point in the patient's When the patient commences palliative care trajectory is a family health history usually taken? When the patient is close to death

After the patient has died

Family health history is not taken Not sure

Other

Please explain

1. In your experience, who usually initiates requests for Patients

DNA testing? Family members

Palliative care health professionals Genetics health professionals Oncology health professionals

It has never been raised in my experience Not sure

Other

Please explain.

7a. Please indicate approximately how often you have been involved in the following activities in the last 12 months.

Zero Once or twice Three to five

times

Six to 10

times

More than 10

times

I have not

worked clinically in the last 12 months

a.

Identifying a patient receiving palliative care who is eligible for

DNA banking/testing.

1. Initiating a discussion about DNA banking/testing with a patient

receiving palliative care or their relative.

1. Seeking advice from a genetics health professional about one of

your palliative patients

1. Referring a patient receiving palliative care to specialist

genetics services.

1. Taking consent for DNA banking/testing from a patient

receiving palliative care.

1. Taking consent for DNA banking/testing from a relative

of a patient receiving palliative care.

7b. Please indicate approximately how often you have been involved in the following activities in the last 12 months.

Zero Once or twice Three to five

times

Six to 10

times

More than 10

times

I have not

worked clinically in the last 12 months

1. Facilitating collection of a DNA sample from a patient receiving

palliative care

1. Disclosing genetic/genomic test results to a patient receiving

palliative care

1. Disclosing a deceased patient's genetic/genomic test results to

bereaved relatives

1. Being aware that I was caring for a palliative patient with an

underlying genetic condition

1. Checking if my palliative patient (or their relatives) had already

had an opportunity to discuss genetics before coming into my care

| 8. | Which scenario best describes the availability of | Specialist genetics services and palliative care |
| --- | --- | --- |
|  | specialist genetics services for your current clinical | services are embedded within the same hospital or |
|  | area? | group of hospitals. |
|  |  | Specialist genetics services and palliative care |
|  |  | services are not embedded within the hospital or |
|  |  | group but are accessible to each other. |
|  |  | Specialist genetics services and palliative care |
|  |  | services are NOT accessible to each other. |
|  |  | Palliative care services have access to private |
|  |  | genetics services only |
|  |  | Other |
|  |  | Not sure |
|  | Please explain |  |

1. In your experience how is consent for DNA banking or Verbal consent only (undocumented) testing obtained and documented? Verbal consent (documented in the patient's

clinical records)

Written consent using locally available paperwork (but not a formal genetics consent form)

Written consent using a formal genetics consent form

In my experience consent has not been taken Other

Please explain.

1. In your experience, what have been the main challenges for palliative care health professionals in

facilitating DNA banking/testing? (Please choose up to 3 responses.)

* Please note: These response options have all been identified as challenges in prior studies. For the purpose of identifying priority areas, we are requesting you nominate your top three challenges

Identifying eligible patients Urgency of the situation/referral

Conflicting priorities between providing palliative care and facilitating genetic testing Obtaining informed consent

Discomfort with initiating discussions about DNA banking/testing with patients or families Palliative care health professionals' lack of

knowledge about DNA banking/testing or procedures Genetics health professionals' lack of knowledge

of the procedure for consent and DNA banking The views or expectations of the family

Lack of availability of specialist genetics services

Communication difficulties between genetics and palliative care services

Conflicting views within the palliative care team about the utility of DNA banking/testing for palliative patients, and their families

Lack of resources

Distress of the patient or family members Complex family dynamics

Concern that a discussion about genetics could damage the therapeutic relationship

Under-referral of palliative patients to genetics services

In my experience DNA banking or genetic/genomic testing has not ever been considered

Other

(You can choose up to 3 responses)

Please explain.

1. Please indicate your level of confidence about the following:

Not at all

confident

Fairly

unconfident

Neither confident

or unconfident

Fairly confident Confident

- 1. Identifying patients who may be

eligible for DNA banking/testing

- 1. Discussing DNA banking with patients or their families
  2. Discussing DNA testing with patients or their families
  3. Contacting my local genetics service
  4. Taking a DNA sample for banking or testing
  5. Disclosing genetic/genomic test results to palliative care patients

Disclosing genetic/genomic test results to bereaved families

- 1. Knowing how to respond if a family member asks me about

their genetic risk

- 1. Knowing my legal responsibilities when sharing

health information with family members when a patient is terminal or after they have died

- 1. Assessing an appropriate time to broach a discussion about

genetics

1. What resources or tools have you found helpful when facilitating DNA banking/testing in the palliative

care setting? (Please indicate all that apply)

Web-based risk assessment tool Smart phone App

Support from a palliative care colleague Contact with specialist genetics services Educational brochures

Telephone information hotline, Face to face education

Online education

Clinical decision-making algorithm Clinical practice guidelines

I have not found any resources or tools helpful Other

How frequently did you receive "face to face

education" for DNA banking/testing in the palliative care setting?

Which clinical practice guidelines did you find useful?

Please explain.

1. What additional resources or tools would be helpful, if any?
2. Please indicate to what extent you agree or disagree with the following statements:

Strongly

disagree

Disagree Neither agree

nor disagree

Agree Strongly agree

Discussing DNA banking/testing with people receiving palliative

care undermines the central ethos of palliative care in providing comfort and support at an emotionally vulnerable time.

- 1. Patients may experience positive emotional benefits from being

able to give a sample for DNA banking/testing for the possible future benefit of their relatives.

- 1. Discussing DNA banking/testing may cause distress to the

families by making them assume/fear their fate is pre-determined.

- 1. DNA banking/testing will have been discussed by other health

professionals before the patient is referred to palliative care.

- 1. The priority in palliative care is to improve quality of life and

relieve suffering and therefore it is not an appropriate time to discuss DNA banking/testing.

- 1. DNA banking/testing of the patient may be important for the

surviving relatives.

- 1. It is not the responsibility of health professionals in palliative

care to discuss DNA banking/testing.

- 1. DNA banking/testing is not appropriate for people receiving

palliative care because it will not help the patient.

1. Please indicate to what extent you agree or disagree with the following statements:
   1. Palliative care health professionals are well placed to have discussions about DNA banking/testing with the family members of a palliative patient

Strongly

disagree

Disagree Neither agree

nor disagree

Agree Strongly agree

Concerns about genetic discrimination (eg. insurance

and employment discrimination) make discussions about DNA banking/testing with palliative patients and their families difficult

- 1. Families generally appreciate being told genetic information

that is relevant to their health

- 1. Discussions about DNA banking/testing need to be

individualised to the palliative patient, and their families

- 1. As time goes on, genetic health information will inevitably

become part of the palliative care health professionals' scope of practice

- 1. If a patient declines a discussion about DNA banking/testing,

palliative care health professionals should revisit this discussion with palliative patients at a later date to see if they've changed their mind

- 1. The family of a palliative patient have a right to know if they are

at risk of developing a genetic disease, regardless of the palliative patient's wishes

- 1. The right of a palliative patient to decline a discussion about

DNA banking/testing are to be respected, regardless of the family's wishes

1. In your opinion, which of these would most help palliative care health professionals to discuss and/or facilitate DNA banking/testing with palliative patients, and their families? (Please choose up to 3 responses)

* Please note: These response options have all been identified as facilitators in prior studies. For the purpose of identifying priority areas, we are requesting you nominate your top three facilitators

Physically co-locating palliative care and genetics health professionals within a hospital or organisation

Developing a specific referral template for palliative care patients to the genetics service that includes relevant family member details Embedding a genetic counsellor in the palliative care team

Having both palliative care and genetics health professionals attending the same multidisciplinary team meetings

Fostering a closer working relationships between palliative care and genetics health professionals Receiving genetics education from genetics health professionals, including ways of sensitively communicating with patients and families about genetics

Policy guidance detailing how and when to discuss DNA banking/testing with palliative patients and their families

Empowering palliative patients, and their families, to seek out DNA banking/testing for themselves

Speaking directly to the genetics health professional about the palliative patient Improving the capability of electronic medical records to share relevant information between health professionals

Collaborating with genetics health professionals to facilitate collection of a DNA sample from a palliative patient

We shouldn't be discussing DNA banking/testing with palliative patients, or their families

Other

Please explain

1. Previous research suggests that patients who are suitable for DNA testing and close to end of life should be offered DNA banking by palliative care health professionals, rather than referring them to the genetics service for DNA testing.

We would like to understand your views about how this would work in practice.

Please indicate your comfort with palliative care health professionals performing the following actions:

- 1. Introduce the idea of DNA banking with the patient and/or the family

Very

uncomfortable

Somewhat

uncomfortable

Neutral Somewhat

comfortable

Very comfortable

- 1. Obtain consent for DNA banking from the patient, or appointed

representative

|  | Facilitate collection of DNA sample |  | |
| --- | --- | --- | --- |
| d. | Organise for the DNA sample to be banked |  |  |
| e. | Instruct the family how to follow up with the genetics service |  |  |
| f. | Communicate family follow up plan to the genetics service |  |  |
|  | Please describe any further thoughts you have about palliative care health professionals performing these actions |  |  |
|  |  |  |  |
|  | Thank you. We will now ask you some demographic questions. |  |  |
| 18. | What is your gender? | Female Male  Self-described (please specify below) Prefer not to say |  |
| 3a | Please describe your gender |  |  |
|  |  |  |  |
| 19. | What is your age group? | < 20  20-24 |  |
|  |  | 25-34 |  |
|  |  | 35-44 |  |
|  |  | 45-54 |  |
|  |  | 55-64  65 or older Prefer to not say |  |
| 20. | What is your country of birth? | Australia New Zealand England India Phillipines Vietnam Italy  South Africa Malaysia Scotland  Other (please specify) Prefer not to say |  |
|  | Please specify your country of birth |  |  |

1. What cultural background or ethnicity do you identify with? (Select all that apply)

None Australian New Zealand English

Irish Scottish Chinese Italian Indian German Greek Vietnamese

Other (please specify)

Prefer not to say

(You may select more than one)

Please describe your cultural background or ethnicity

1. Which language do you mainly speak at home? (If more English than one language, indicate the one that is spoken Mandarin

most often) Arabic

Cantonese Vietnamese Italian Greek

Hindi Spanish Punjabi

Other (please specify) Prefer not to say

Please describe the language you mostly speak at home

1. What is your primary profession? Medical Nursing Other

Please explain.

1. How many years has it been since you qualified in your Less than 2 years current profession? 2 to 5 years

6 to 10 years

11 to 15 years More than 15 years

1. What is your highest academic achievement? PhD

Master degree Bachelor degree Diploma

Professional qualification Other

Please explain.

1. What is your primary area of specialty? Oncology Palliative care Primary care Aged care Other

Please explain.

1. Which best describes your job title? Registered nurse

Senior registered nurse (including nurse practitioner)

Junior doctor (eg intern, resident or equivalent) Senior doctor (eg registrar, fellow or equivalent) Consultant doctor (including staff specialist or equivalent)

Other (please specify)

Please explain.

1. How long have you worked in your current specialist Less than 2 years area? 2 to 5 years

6 to 10 years

11 to 15 years More than 15 years

1. Which country do you work in? Australia New Zealand England Scotland

Northern Ireland Wales

Other

Please explain.

1. Which sector do you usually work in? Public Private

Public and private Other

Please explain.

1. What location do you usually work in? City/metropolitan/urban Regional

Rural Other

Please explain.

1. What setting do you usually work in? Hospital Hospice

Community clinic Home care General Practice Other

Please explain.

**Many thanks for your participation in this survey.**

**If you have any further comments, you may write them below. When you are finished, please press submit.**

**Have a great day!**

1. Please add any further comments about facilitating DNA banking/testing with people receiving palliative care

or their families.

1. Please tell us if you have any further comments about this survey or study.

# Appendix C: Participant Information Sheet (Survey Landing Page)

**Investigating the barriers and facilitators to genetic/genomic testing for people receiving palliative care in Australia, New Zealand and the United Kingdom**

[UTS HREC REF NO. ETH19-2408/21-5854]

**What is the research study about?**

The purpose of this research/online survey is to investigate the existing facilitators and barriers to genetic/genomic testing in the adult palliative care setting.

You have been invited to participate because you are a member of a professional organisation of healthcare professionals specialising in either palliative care, genetics or genomics. We are interested in understanding your experiences and views of discussing genetics with people who have palliative care needs, and their families.

**Who is conducting this research?**

My name is Stephanie White and I am a PhD candidate at UTS. The research team includes experts in genetic counselling, clinical genetics, palliative care medicine and nursing and medical ethics.

**Other members of our study team:**

Dr Chris Jacobs, Senior Lecturer, Genetic Counselling, UTSAssociate Professor Alison McEwen, Head of Genetic Counselling, UTSProfessor Jane Phillips, Professor of Palliative Care Nursing, UTSAssociate Professor Kathy Tucker, Consultant Clinical Geneticist, Prince of Wales Hospital (POWH), SydneyDr Erin Turbitt, Lecturer, Genetic Counselling, UTSDr Megan Best, Associate Professor of Bioethics, Institute for Ethics and Society, The University of Notre DameDr April Morrow, Genetic Counsellor, POWH, SydneyEligibility Criteria

You have been invited to participate because you work or have worked as a palliative care or genetics health professional. Please do not complete this survey if you have never worked as a health care professional in palliative care or genetics/genomics.

**Do I have to take part in this research study?**

Participation in this study is voluntary. It is completely up to you whether or not you decide to take part.

If you decide to participate, you will be directed to complete an online survey that will take 10 to 20 minutes of your time. You will be asked some demographic questions, such as about your professional and clinical area, genetics/genomics education and views and experiences of DNA banking/testing in adult palliative care. Please answer all the questions that you are directed to. If you receive the survey from more than one organisation, please only complete it once.

You can change your mind at any time and stop completing the survey without consequences.

**Are there any risks/inconvenience?**

It is possible that the questionnaire could cause upset by reminding participants of an uncomfortable experience of raising a difficult issue with patients or families or leads to feelings of embarrassment or regret at not having discussed genetic testing with potentially eligible patients or families. If you experience feelings of distress as a result of participation in this study you can let the researcher know and they will provide you with assistance.

Alternatively, please contact your usual workplace support provider, occupational health service, or General Practitioner. Accessible, anonymous support is also available from support organisations as follows: In the UK, the number for Samaritans is 116 123 In Australia, the number for Lifeline is 13 11 44 In New Zealand, the number for Lifeline is 0800 543 354

**What will happen to information about me?**

Access to the online questionnaire is online through this link. Submission of the online questionnaire/s is an indication of your consent. By responding "Yes" to "I have read the information above and I agree to taking part in this survey", you consent to the research team collecting and using personal information about you for the research project. All this information will be treated confidentially. The data will be securely stored on a password protected university computer with access limited to the research team. Your information will only be used for the purpose of this research project, except as required by law.

We plan to publish results in a peer-reviewed academic journal, and to disseminate findings to health professionals, patient groups and policy makers through conference presentations and publications. Results from this research will be included in Stephanie White's thesis. In any publication, information will be provided in such a way that you cannot be identified.

**What if I have concerns or a complaint?**

If you have concerns about the research that you think the researcher can help you with, please feel free to contact Stephanie White by email at stephanie.white@uts.edu.au

If you would like to talk to someone who is not connected with the research, you may contact: UTS Research Ethics Officer

Phone: 02 9514 9772 E-mail: research.ethic@uts.edu.au And quote UTS HREC REF NO. ETH18-2408.

This study has been reviewed by and received ethics clearance through University of Technology Sydney's Human Research Ethics Committee.

If you've received this survey from more than one organisation, please only complete it once.

Please choose which group of health professions your Palliative care health professionals occupation would fall under. You will then be directed Genetics health professionals

to the relevant survey. None of the above

I have read the information above and I agree to Yes

taking part in this survey No

# Appendix D: Full List of Barriers, Facilitators, and Resources/Tools

| Table A. Participants were asked to select their top 3 challenges (barriers) to integrating genetics into the care of people with palliative care needs and their families. | | | | | | | |
| --- | --- | --- | --- | --- | --- | --- | --- |
| Challenge descriptions | TOTAL (n=72) | | G-HP (n=29) | | PC-HP (n=43) | | p-value |
|  | **n** | **%** | **n** | **%** | **n** | **%** |  |
| Palliative care HPs’ lack of knowledge | 32 | 44 | 13 | 45 | 19 | 44 | 1 |
| Identifying eligible patients* | 19 | 26 | 4 | 14 | 15 | 35 | 0.046# |
| Conflicting priorities between providing palliative care and genetic testing | 15 | 21 | 6 | 21 | 9 | 21 | 1 |
| Under-referral of palliative patients to genetics* | 15 | 21 | 12 | 41 | 3 | 7 | <0.001# |
| Urgency of the situation/referral | 13 | 18 | 8 | 28 | 5 | 12 | 0.119 |
| In my experience DNA storage or testing has not been considered* | 11 | 15 | 1 | 3 | 10 | 23 | 0.041# |
| Discomfort with initiating DNA storage/testing discussions | 10 | 14 | 6 | 21 | 4 | 9 | 0.187 |
| Obtaining informed consent | 6 | 8 | 4 | 14 | 2 | 5 | 0.212 |
| Conflicting views within the palliative care team about DNA banking/testing* | 6 | 8 | 5 | 17 | 1 | 2 | 0.036# |
| The views or expectations of the family | 5 | 7 | 4 | 14 | 1 | 2 | 0.15 |
| Communication between genetics and palliative care services* | 5 | 7 | 5 | 5 | 0 | 0 | 0.008# |
| Distress of the patient or family members | 5 | 7 | 2 | 7 | 3 | 7 | 1 |
| Lack of specialist genetics services | 4 | 6 | 0 | 0 | 4 | 9 | 0.143 |
| Lack of resources | 4 | 6 | 2 | 7 | 2 | 5 | 1 |
| Complex family dynamics | 4 | 6 | 0 | 0 | 4 | 9 | 0.143 |
| Other | 3 | 4 | 1 | 3 | 2 | 5 | 1 |
| Concerns about harming the therapeutic relationship | 1 | 1 | 0 | 0 | 1 | 2 | 1 |
| Genetics HPs’ lack of knowledge | 0 | 0 | 0 | 0 | 0 | 0 | NA |

| Table B. Participants were asked to select their top 3 facilitators to integrating genetics into the care of people with palliative care needs and their families. | | | | | | | |
| --- | --- | --- | --- | --- | --- | --- | --- |
| Facilitator description | TOTAL (n=72) | | G-HPs (n=29) | | PC-HPs (n=43) | | **p-value** |
|  | **n** | **%** | **n** | **%** | **n** | **%** |  |
| Developing a specific genetic referral template for palliative care patients | 31 | 43 | 9 | 31 | 22 | 51 | 0.145 |
| Fostering closer working relationships between palliative care & genetics health professional | 27 | 38 | 15 | 52 | 12 | 28 | 0.05 |
| Genetics health professionals deliver education to palliative care health professionals | 25 | 35 | 11 | 38 | 14 | 33 | 0.801 |
| Embedding a genetic counsellor in the palliative care team | 17 | 24 | 8 | 28 | 9 | 21 | 0.578 |
| Palliative care & genetics health professional attend the same multidisciplinary team meetings* | 15 | 21 | 11 | 38 | 4 | 9 | 0.006 |
| Policy guidance for discussing DNA banking/testing | 12 | 17 | 6 | 21 | 6 | 14 | 0.455 |
| Physically co-locating palliative care and genetics services | 8 | 11 | 2 | 7 | 6 | 14 | 0.461 |
| Empowering palliative patients and families to seek out DNA banking/testing for themselves | 7 | 10 | 1 | 3 | 6 | 14 | 0.23 |
| Speaking directly to the palliative care health professional about the palliative patient | 7 | 10 | 2 | 7 | 5 | 12 | 0.694 |
| Collaborating with genetics health professional to facilitate collection of a DNA sample | 7 | 10 | 5 | 17 | 2 | 5 | 0.11 |
| Improving electronic medical record capabilities | 2 | 3 | 1 | 3 | 1 | 2 | 1 |
| Other | 2 | 3 | 0 | 0 | 2 | 5 | 0.512 |
| We shouldn't be discussing DNA banking / testing with palliative patients, or their families | 1 | 1 | 0 | 0 | 1 | 2 | 1 |

| Table C. Participants were asked to select the most useful resources or tools to support them to integrate genetics into the care of people with palliative care needs and their families. | | | | | | |
| --- | --- | --- | --- | --- | --- | --- |
| Resources or tools to support HPs to facilitate DNA banking or testing | TOTAL (n=72) | | G-HP (n=29) | | PC-HP (n=43) | |
|  | **n** | **%** | **n** | **%** | **n** | **%** |
| Support from a specialist genetics service or colleague | 33 | 46 | 19 | 66 | 14 | 33 |
| Support from a palliative care colleague | 15 | 21 | 9 | 31 | 6 | 14 |
| I have not found any resources or tools helpful | 10 | 14 | 3 | 10 | 7 | 16 |
| Other/no experience | 10 | 14 | 1 | 3 | 9 | 21 |
| Clinical decision-making algorithm or guideline | 9 | 13 | 3 | 10 | 6 | 14 |
| Web-based risk assessment tool | 7 | 10 | 4 | 14 | 3 | 7 |
| Educational brochures | 6 | 8 | 2 | 7 | 4 | 9 |
| Face to face or online education | 5 | 7 | 2 | 7 | 3 | 7 |
| Telephone information hotline | 3 | 4 | 1 | 3 | 2 | 5 |
| Smart phone App | 1 | 1 | 0 | 0 | 1 | 2 |
